# Association of diabetes, hypertension, and their combination with basal symptoms and treatment responses in overactive bladder patients

**DOI:** 10.3389/fphar.2023.1144470

**Published:** 2023-03-30

**Authors:** A. Elif Müderrisoglu, Ayse A. Sakul, Sandra Murgas, Jean J. M. C. H. de la Rosette, Martin C. Michel

**Affiliations:** ^1^ Department of Medical Pharmacology, Istanbul Medipol University, Istanbul, Türkiye; ^2^ APOGEPHA Arzneimittel GmbH, Dresden, Germany; ^3^ Department of Urology, Medipol Mega University Hospital, Istanbul Medipol University, Istanbul, Türkiye; ^4^ Department of Pharmacology, University Medical Center, Johannes Gutenberg University, Mainz, Germany

**Keywords:** overactive bladder syndrome, diabetes, hypertension, comorbidity, propiverine, treatment, non-interventional study

## Abstract

**Introduction:** Pelvic hypoperfusion caused by atherosclerosis has been proposed as a cause of lower urinary tract dysfunction including overactive bladder syndrome (OAB). Limited data indicate that OAB patients with concomitant diabetes or hypertension, known risk factors of atherosclerosis, may exhibit greater baseline OAB symptoms and slightly smaller therapeutic responses to treatment, but the impact of a combined presence of diabetes and hypertension has not been reported. Therefore, we have explored whether the combined presence of both comorbidities is associated with greater baseline OAB symptoms than that of either comorbidity alone. Secondary questions were exploration of the impact of either comorbidity on baseline symptoms, and of the impact of either comorbidity alone and their combination on therapeutic responses.

**Methods:** Data from two non-interventional studies applying treatment with propiverine ER 30 or 45 mg/d for 12 weeks were analyzed.

**Results:** Number of urgency episodes in the combination group was greater than with each comorbidity alone. The impact of comorbidities on baseline intensity of incontinence, frequency or nocturia or Patient Perception of Bladder Condition was less consistent or absent. Either comorbidity alone was associated with a smaller % improvement of symptoms, and their combination had a greater effect than either alone. However, all attenuations associated with comorbidity were small relative to the overall improvement.

**Conclusions:** We conclude that comorbidities of diabetes and hypertension have detectable effects on OAB symptoms and treatment responses, but the small magnitude of these alterations does not justify changing existing paradigms for the clinical management of OAB.

## 1 Introduction

Lower urinary tract dysfunction in general and the overactive bladder syndrome (OAB) in particular are highly prevalent conditions in the general population (Milsom et al., 2001). An emerging theory proposes that chronic pelvic hypoperfusion driven by atherosclerosis may contribute to the genesis and pathophysiology of lower urinary tract dysfunction including OAB (Michel et al., 2015; Thurmond et al., 2016). Diabetes and hypertension are key risk factors for atherosclerosis (Poznyak et al., 2022). The findings from several clinical studies support this hypothesis in men with lower urinary tract symptoms (LUTS) attributed to benign prostatic hyperplasia (BPH) (Michel et al., 2000; Joseph et al., 2003; Michel et al., 2004b; Rohrmann et al., 2005; Gupta et al., 2006; Ponholzer et al., 2006; Martin et al., 2011), although some studies did not (Meigs et al., 2001; Gupta et al., 2006; Martin et al., 2011). For instance, the concomitant presence of diabetes was associated with an 1.9 point greater International Prostate Symptom Score (IPSS), a 1.1 mL/s smaller maximum urinary flow rate, and an 8.9 mL greater post-void residual in a cohort of 9,856 men (Michel et al., 2000). Analyzing the same database, concomitant presence of hypertension was associated with an 1.3–2.1 points greater IPSS (depending on definition of hypertension) and a 0.9 mL/s smaller maximum urinary flow rate (Michel et al., 2004b). Similarly, subjects with concomitant diabetes had a worse nocturia quality of life score than those without diabetes in a study of 5,775 men with LUTS attributed to BPH (Michel et al., 2020c). The presence of diabetes (Fu et al., 2016) or hypertension was also reported to be associated with a greater progression of male LUTS (Marshall et al., 2014; Fu et al., 2016).

An association of OAB with diabetes and/or metabolic syndrome has also been shown in various clinical studies (Martin et al., 2011; Golabek et al., 2012; Bunn et al., 2015; Zhang et al., 2015) although a systematic review of such studies concluded that the evidence was limited and often based on studies of poor quality (Bunn et al., 2015). On the other hand, the presence of diabetes has more consistently been linked to bladder dysfunction in more than 100 studies in experimental animals (Arioglu-Inan et al., 2018; Ellenbroek et al., 2018; Yesilyurt et al., 2022). While one large population-based study has demonstrated an association of OAB with hypertension (Zhang et al., 2015), evidence for an association of OAB with hypertension largely relies on studies in experimental animals that have consistently linked hypertension to an OAB-like phenotype (Persson et al., 1998; Spitsbergen et al., 1998; Clemow et al., 1999; Steers et al., 1999; Monica et al., 2008; Ramos-Filho et al., 2011).

Interestingly, the atherosclerosis key risk factors diabetes and hypertension often coexist (Poznyak et al., 2022). Such coexistence may have greater effects on atherosclerosis than either condition alone. However, the effect of the combined presence of diabetes and hypertension on OAB has not been explored to our knowledge. A large population-based study has applied multivariate analysis to demonstrate that the likelihood of having bothering LUTS in women was increased independently by both diabetes and hypertension (Zhang et al., 2015) but did not assess the impact of concomitant presence.

A related but distinct question is whether patients with OAB and concomitant diabetes or hypertension exhibit greater symptom severity and/or altered responses to treatment with OAB medications. This has been addressed in a limited number of studies among OAB patients with concomitant diabetes (Schneider et al., 2013) or hypertension (Michel et al., 2022). Based on the pelvic hypoperfusion/atherosclerosis hypothesis of OAB (Michel et al., 2015; Thurmond et al., 2016) and the role of both diabetes and hypertension as risk factors for atherosclerosis and their frequent co-existence (Poznyak et al., 2022), it can be expected that the combined presence of both comorbid conditions should have a greater impact on OAB than either condition alone. However, little clinical data are available in this regard. There is also limited data on the question whether the combined presence of diabetes and hypertension affects therapeutic outcomes in OAB patients (Erdogan et al., 2022). Therefore, we have used data from two large non-interventional studies in OAB patients (Amiri et al., 2021) to explore the association of diabetes, hypertension and their combination with baseline symptoms of OAB patients and their effect on therapeutic outcomes in a clinically meaningful way under real-world conditions. In this regard, the primary question was how the combination affects baseline OAB symptoms, whereas the effect of diabetes and hypertension alone on baseline symptoms and the effect of either comorbidity alone or of their combination on treatment outcomes were secondary questions.

## 2 Patients and methods

Our analyses are based on the data from two previously published non-interventional studies (Amiri et al., 2021). Each study had been approved by the Ethics Committee of State Board of Physicians of Saxony, Germany (Sächsische Landesärztekammer, Dresden, Germany; EK-BR-14/12-1 and EK-BR-18/14-1). The underlying studies were performed in accordance with the Helsinki Declaration of 1964, and its later amendments. The additional analyses of the databases reported here were additionally approved by the ethical committee of Medipol University in Istanbul, Turkey (E-10840098-772.02-4785). While the planned explorations constitute *post-hoc* analyses, they are based on a protocol that had been finalized and published (doi 10.17605/OSF.IO/TQDN7) before any data relative to the study questions were viewed.

Full details of the two underlying studies have been reported (Amiri et al., 2021). Briefly, patients with OAB who started treatment with 30 or 45 mg propiverine per day in its extended-release formulation based on physician judgement were systematically followed for an intended observation period of about 12 weeks. In accordance with the non-interventional character of the studies, no inclusion or exclusion characteristics were specified other than those defined in the applicable summary of product characteristics. Study I consisted of 1,335 and study II of 745 patients. The case record form for each patient had explicitly asked for the concomitant presence of diabetes and hypertension, which was used for classification for the present analyses. In line with the non-interventional character of the studies, participating physicians were given no instructions on how to diagnose either comorbidity. In line with the non-interventional character of the study, the protocol did not specify whether OAB-related data were collected from voiding diaries or from patient recollection, but the applicable German guideline at the time the studies were performed recommended use of voiding diaries (Dimpfl et al., 2010).

The planned analyses largely followed those of a previous publication comparing OAB patients with and without diabetes treated with darifenacin (Schneider et al., 2013). For the purpose of the current analysis, four groups of patients were defined:

- Those with neither reported diabetes nor hypertension (control group)
- Those with reported diabetes but no reported hypertension (diabetic group)
- Those with reported hypertension but not reported diabetes (hypertensive group)
- Those with reported concomitant diabetes and hypertension (combination group)

In line with our previous analyses of this database (Amiri et al., 2021), medically implausible values were ignored in the analyses. We defined these as >50 urgency, >30 incontinence, >40 frequency, and >20 nocturia episodes per 24 h.

For each of the four groups, a descriptive analysis was performed for the following categorical (gender, prescribed dose at study end), ordinal (Patient Perception of Bladder Condition (PPBC) score) and continuous parameters (all others) at baseline:

- Gender
- Age
- Height
- Body weight
- Body mass index
- Number of concomitant medications
- Number of micturitions per 24 h
- Number or urgency episodes per 24 h (only in those reporting at least 1 episode at baseline)
- Number of incontinence episodes per 24 h (only in those reporting at least 1 episode at baseline)
- Number of nocturia episodes per 24 h (only in those reporting at least 1 episode at baseline)
- PPBC score

After 12 weeks of treatment, the following treatment-associated changes were assessed. Based on our previous analyses (Amiri et al., 2020), they were calculated as % changes relative to baseline, except for PPBC which is shown as distribution diagrams (Schönburg et al., 2022).

- % change in number of micturitions per 24 h
- % change in number or urgency episodes per 24 h
- % change in number of incontinence episodes per 24 h
- % change in number of nocturia episodes per 24 h

Finally, based on other previous analyses (Müderrisoglu et al., 2022), we have calculated how many patients became symptom-free in each group. Becoming symptom-free was defined as 0 episodes of urgency and incontinence and as <8 micturitions per day. For nocturia, two parallel definitions were applied: 0 episodes in all patients having at least 1 episode of nocturia at baseline, and ≤1 episodes in all patients having at least 2 episodes of nocturia at baseline.

As many of the parameters in question exhibit a non-normal distribution (Amiri et al., 2020), data are presented as medians with interquartile ranges, except for categorical variables that are presented as percentage of n patients and ordinal variables presented as distribution curves. To enable better comparison with published data from other studies, means ± SD are also reported. Our analysis and reporting for urgency, incontinence, micturition frequency, and nocturia was restricted to those exhibiting that symptom at baseline, which means that, e.g., patients not exhibiting incontinence were not included in the calculation of median number of incontinence episodes; the percentage of patients not exhibiting a given symptom is indicated.

Descriptive statistical analysis was performed with GraphPad Prism 9.3 or higher (Los Angeles, CA, United States). In line with the exploratory character of the analyses and with recommendations by leading statisticians (Amrhein et al., 2019; Michel et al., 2020a), no hypothesis-testing statistical analyses were performed. Because of the exploratory character of our analysis, multiple comparison adjustments were not applied. However, we consider the concomitant analysis of two studies of similar design to potentially support robustness of the findings. Rather we focused on the question whether observed effect sizes were likely to be clinically relevant.

## 3 Results

### 3.1 Demographics and baseline values

#### 3.1.1 Study I

In study I, 691, 114, 370, and 160 subjects were in the control, diabetic, hypertensive and combination group, respectively. Women were more frequent in the control and hypertension than in the diabetes and combination groups (Table 1). Median age was lowest in the control group, higher in diabetic and hypertensive patients, and numerically highest in the patients, the latter accounting for an age difference compared to the control group of 10 years. Height was comparable across all four groups (Table 1). In contrast, body weight and BMI was lowest in control patients and highest in combination patients (Table 1). The median reported number of comedications was 0 in the control group, 1 in the diabetes and hypertension group, and 2 in the combination group (Table 1). While subjects without reported diabetes or hypertension had no reported anti-diabetic or anti-hypertensive comedication, 80.7% in the diabetic group had at least one anti-diabetic comedication, 87.0% in the hypertensive group at least one anti-hypertensive comedication, and 80.0% in the combination group at least one anti-diabetic and at least one anti-hypertensive comedication.

**TABLE 1:** Demographics and baseline data from study I Data are shown as medians with inter-quartile ranges and as means ± SD in parentheses except for categorical variables that are shown as % of cohort. Data on baseline symptoms are shown only for those exhibiting a pathological value at baseline.

	Control	Diabetes	Hypertension	Combination
Gender, % females	68.3	63.1	66.3	59.6
Age, years	63 [52; 73]	70 [62; 76]	72 [64; 77]	73 [66; 77]
	61.7 ± 14.3	68.3 ± 10.8	70.1 ± 9.6	71.4 ± 8.4
Height, cm	169 [164; 175]	170 [165; 177]	168 [162; 175]	170 [165; 176]
	169 ± 8	171 ± 8	168 ± 8	170 ± 8
Body weight, kg	74 [67; 83]	80 [71; 89]	79 [70; 85]	83 [75; 92]
	75.1 ± 13.2	81.6 ± 15.9	78.7 ± 14.5	85.9 ± 17.2
BMI, kg/m^2^/1.73	26 [24; 28]	27 [25; 30]	28 [25; 30]	29 [26; 33]
	26.2 ± 3.9	28.0 ± 4.6	27.8 ± 4.6	29.8 ± 5.3
Comedications, number	0 [0; 0]	1 [1; 1]	1 [1; 2]	2 [2; 3]
	0.31 ± 0.65	1.21 ± 0.99	1.59 ± 1.17	2.59 ± 1.63
Urgency episodes/24 h	9 [6; 12]	10 [6; 13]	9 [6; 14]	12 [6; 15]
	9.7 ± 5.7	9.9 ± 5.1	10.2 ± 6.2	11.3 ± 5.5
Incontinence episodes/24	4 [2; 6]	3 [2; 5.3]	4 [2; 7]	5 [3; 8]
	4.8 ± 3.8	4.4. ± 3.2	4.8 ± 3.8	6.2 ± 4.4
Voids/24 h	13 [11; 16]	13 [12; 16]	13 [11; 16]	14 [12; 17]
	13.6 ± 3.9	13.9 ± 3.7	13.9 ± 4.0	14.6 ± 4.0
Nocturia episodes/24 h	3 [2; 4]	4 [3; 4.5]	3 [2; 4]	4 [3; 4]
	3.4 ± 1.7	3.6 ± 1.4	3.5 ± 1.7	3.5 ± 1.4
PPBS score, rank	5 [4; 5]	5 [4; 5]	5 [4; 5]	5 [4; 5]
	4.6 ± 0.8	4.7 ± 0.7	4.7 ± 0.8	4.8 ± 0.7

**FIGURE 1:**
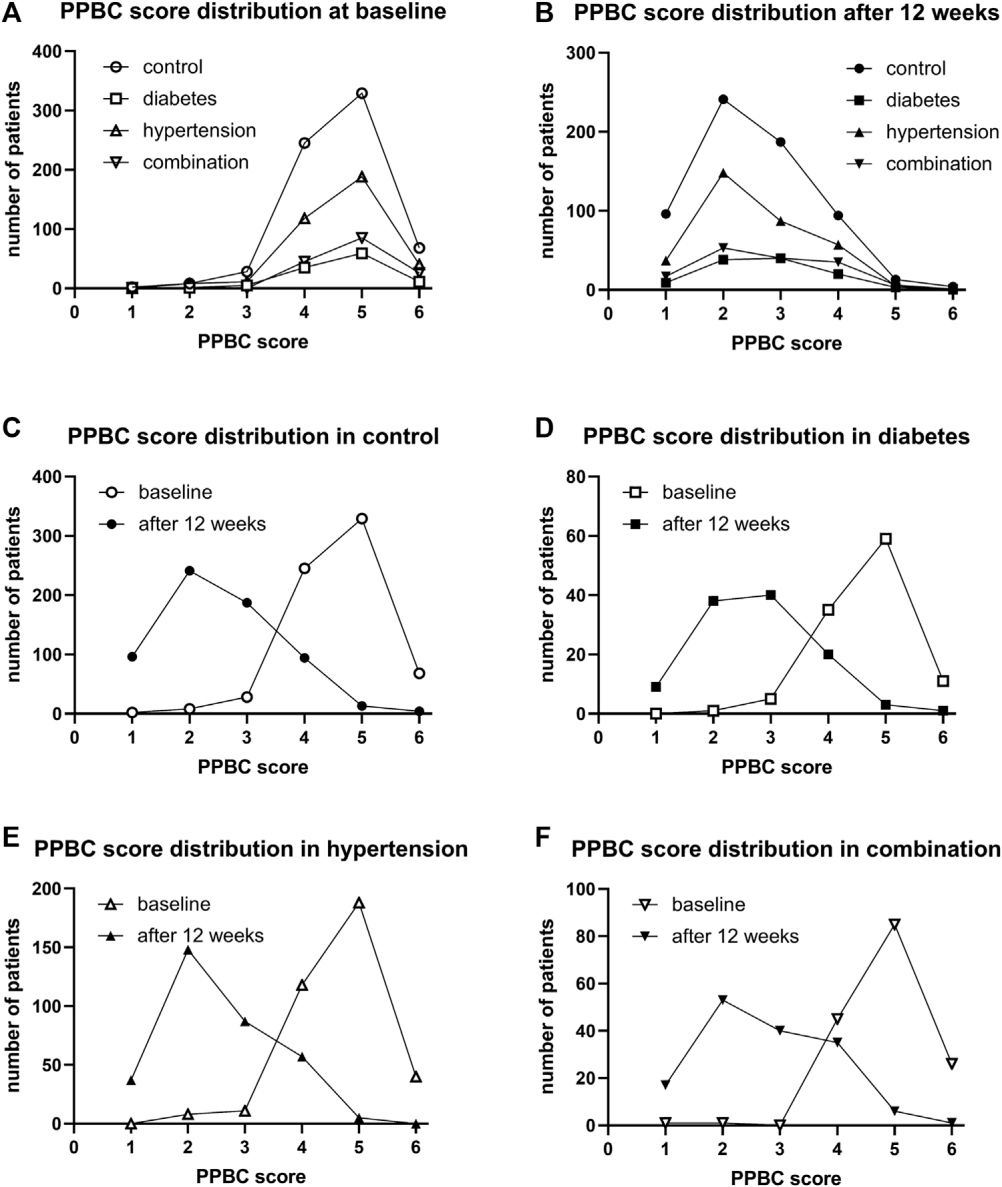
Frequency distribution of the PPBC score in study I: **(A)** across groups at baseline, **(B)** across groups after 12 weeks of treatment, **(C)** comparison of baseline and after 12 weeks of treatment in control patients, **(D)** comparison in diabetic patients, **(E)** comparison in hypertensive patients, and **(F)** comparison in patients with both comorbidities.

Urgency was present in 98.8%, 98.2%, 98.1%, and 99.4% of all subjects in the four groups. Incontinence was present in 68.2%, 71.6%, 72.0%, 82.5%. A micturition frequency >7 per 24 h was reported by 94.6%, 99.1%, 94.0%, and 97.5%. Nocturia (at least 1 episode) was found in 94.7%, 97.3%, 97.6%, and 99.4%. In those expressing a given symptom, the median basal episode number was mostly lowest in the control and highest in the combination group; however, except for urgency episodes, differences between groups were small (Table 1). The median PPBC score was 5 [95% confidence interval 4; 5] in all groups (Table 1), and its distribution was also similar across groups (Figure 1).

#### 3.1.2 Study II

In study II, 345, 62, 227, and 111 subjects were in the control, diabetic, hypertensive and combination group, respectively. Their demographic characteristics are shown in Table 2 and were generally similar as those in study I, including trends for differentiation between groups. While subjects without reported diabetes or hypertension had no reported anti-diabetic or anti-hypertensive comedication, 82.3% in the diabetic group had at least one anti-diabetic comedication, 84.1% in the hypertensive group at least one anti-hypertensive comedication, and 84.7% in the combination group at least one anti-diabetic and at least one anti-hypertensive comedication.

**TABLE 2:** Demographics and baseline data from study II Data are shown as medians with inter-quartile ranges and as means ± SD in parentheses except for categorical variables that are shown as % of cohort. Data on baseline symptoms are shown only for those exhibiting a pathological value at baseline.

	Control	Diabetes	Hypertension	Combination
Gender, % females	62.3	60.7	62.8	62.3
Age, years	64 [51; 73]	70 [64; 76.5]	71 [65; 75]	72 [67; 77]
	61.5 ± 14.9	68.6 ± 10.5	69.5 ± 10.0	71.2 ± 9.0
Height, cm	170 [164; 175]	170 [164.8; 176]	169 [164; 176]	169 [164.3; 175]
	169.8 ± 7.6	170.2 ± 7.7	169.4 ± 8.3	169.6 ± 7.8
Body weight, kg	76 [67; 84]	82.5 [73; 90.5]	80 [70; 86]	81 [75; 81.3]
	75.9 ± 14.4	84.4 ± 16.5	79.3 ± 14.0	83.1 ± 13.5
BMI, kg/m^2^/1.73	26.1 [23.7; 28.3]	28.5 [26.7; 31.1]	27.1 [25.0; 29.4]	28.1 [26.3; 31.1]
	26.4 ± 4.5	29.1 ± 4.8	27.6 ± 4.2	28.9 ± 4.1
Comedications, number	0 [0; 1]	1 [1; 1]	1 [1; 2]	2 [2; 3]
	0.36 ± 0.64	1.10 ± 0.69	1.67 ± 1.38	2.85 ± 1.655 [4; 5]
Urgency episodes/24 h	9 [5.8; 12]	9 [7; 14]	9 [5; 12]	10 [6; 13]
	9.3 ± 5.1	10.8 ± 7.3	9.2 ± 5.0	10.3 ± 5.9
Incontinence episodes/24	4 [2; 6]	5 [3; 7.5]	4 [2; 7]	5 [3; 7]
	4.8 ± 3.5	5.7 ± 4.0	5.2 ± 4.1	5.6 ± 4.0
Voids/24 h	13 [11; 15]	14.5 [11; 18]	13 [10; 15]	14 [12; 17]
	13.4 ± 3.9	14.7 ± 4.3	13.2 ± 3.7	14.6 ± 4.4
Nocturia episodes/24 h	3 [2; 4]	4 [3; 5]	3 [2; 4]	3 [2.5; 4]
	3.4 ± 1.7	4.0 ± 1.9	3.4 ± 1.6	3.6 ± 1.6
PPBS score, rank	5 [4; 5]	5 [4; 5]	5 [4; 5]	5 [4; 5]
	4.52 ± 0.81	4.80 ± 0.87	4.52 ± 0.91	4.51 ± 0.89

**FIGURE 2:**
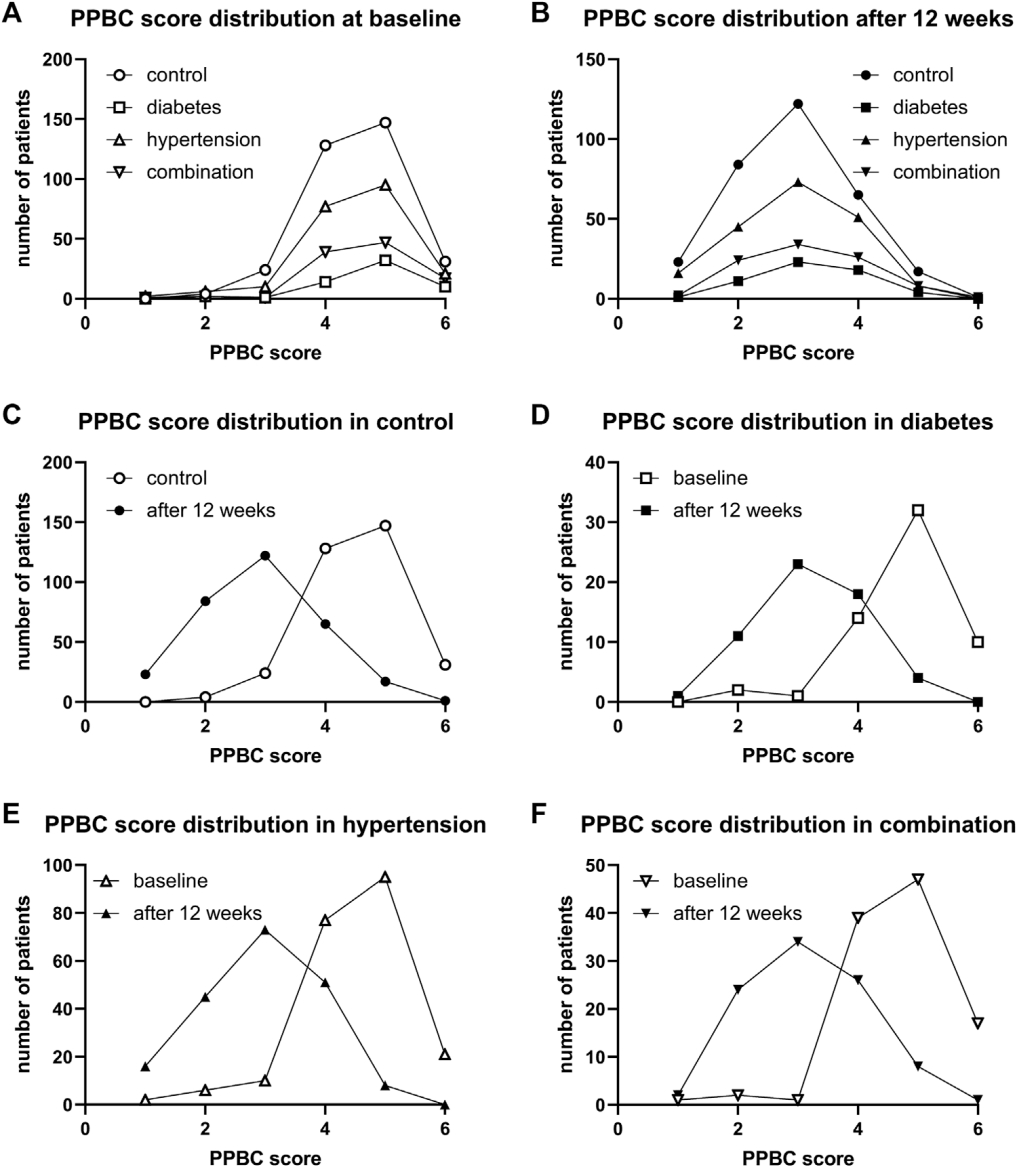
Frequency distribution of PPBC score in study II: **(A)** across groups at baseline, **(B)** across groups after 12 weeks of treatment, **(C)** comparison of baseline and after 12 weeks of treatment in control patients, **(D)** comparison in diabetic patients, **(E)** comparison in hypertensive patients, and **(F)** comparison in patients with both comorbidities.

Urgency was present in 97.0%, 100%, 97.7%, and 98.1% of all subjects in the four groups. Incontinence was present in 71.4%, 80.0%, 73.6%, 83.3%. A micturition frequency >7 per 24 h was reported by 94.2%, 96.8%, 92.0%, and 90.1%. Nocturia (at least 1 episode) was found in 96.1%, 93.6%, 97.8%, and 99.1%. In those expressing a given symptom, the median basal episode number was highest in the combination group for urgency and incontinence although not necessarily greater than in the other groups. Frequency and nocturia were highest in the diabetes group. However, all differences between groups were small (Table 2). The median PPBC score was 5 [4; 5] in all groups, and its distribution was also similar across groups (Figure 2).

### 3.2 Treatment responses

#### 3.1.3 Study I

**FIGURE 3:**
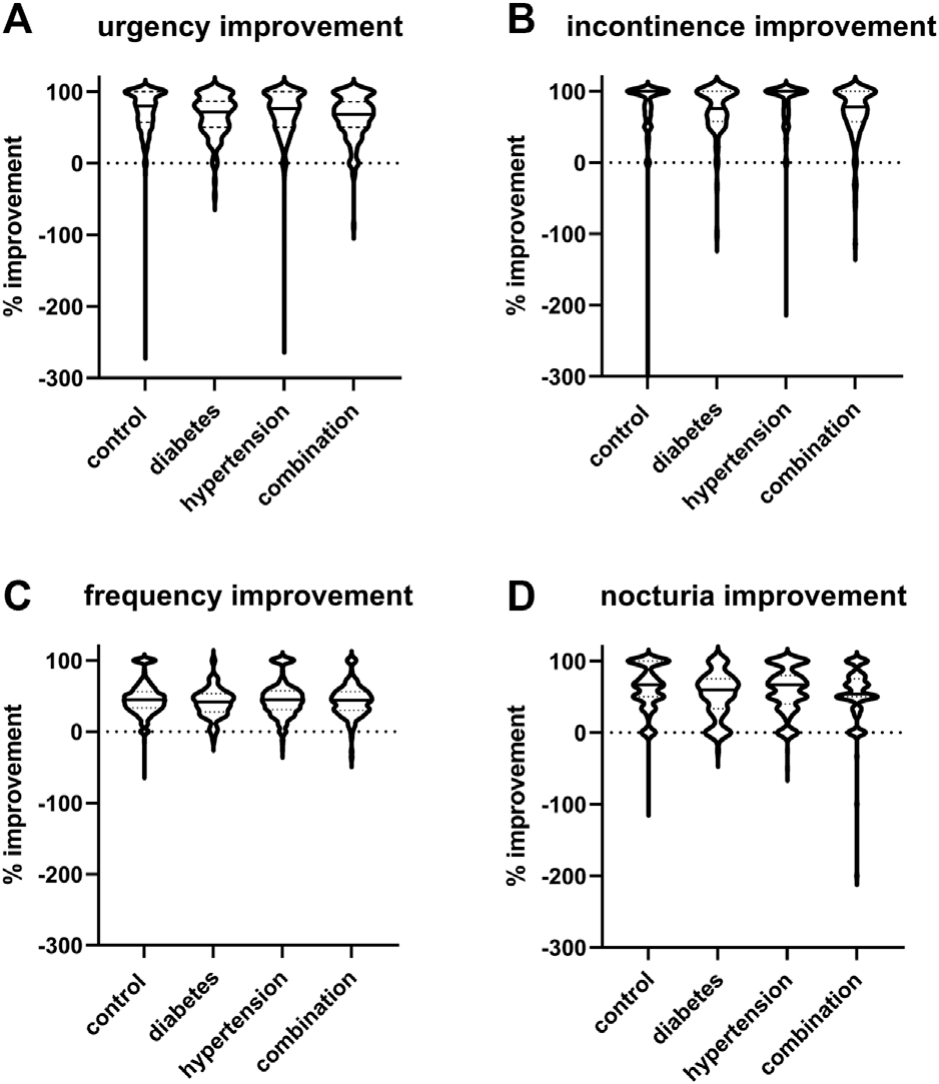
Violin plots of % changes in study I in number of **(A)** urgency, **(B)** incontinence, **(C)** frequency, and **(D)** nocturia episodes per 24 h. Median and interquartile ranges are indicated by the horizontal solid and dashed lines, respectively, within each plot. Note that only subject with a pathological value at baseline were included a very small number of subjects exhibited a worsening, indicated here as negative value.

The mean reduction in urgency episodes was 72.8% ± 32.0% in control patients, 66.0% ± 29.2% in diabetic patients, 69.5% ± 35.7% in hypertensive patients, and 64.0% ± 30.8% in those with both comorbidities (median values with interquartile ranges shown in Figure 3). Similarly, the mean reduction in incontinence episodes was 77.3% ± 37.6% in control patients, 73.1% ± 34.5% in diabetic patients, 81.4% ± 33.9% in hypertensive patients, and 69.4% ± 37.2% in those with both comorbidities (Figure 3). The mean reduction in number of micturitions was 45.9% ± 22.9% in control patients, 40.5% ± 20.0% in diabetic patients, 45.5% ± 24.5% in hypertensive patients, and 43.5% ± 22.4% in those with both comorbidities (Figure 3). Finally, the mean reduction in nocturia episodes was 62.9% ± 30.7% in control patients, 52.8% ± 31.6% in diabetic patients, 59.6% ± 30.7% in hypertensive patients, and 54.0% ± 38.0% in those with both comorbidities (Figure 3). Becoming free of urgency was reported most often in control (30.8%), less often in hypertension and least in diabetes or combination (Table 3). Becoming free of incontinence and nocturia (control: 52.5% and 18.8%, respectively) exhibited a similar picture. While the situation was also similar for frequency, differences between groups were smaller than for the other three symptoms.

**TABLE 3:** Patients becoming free of a given symptom upon treatment in study I Data are shown as % of patients who had exhibited a symptom at baseline but did not at end of study. Not exhibiting a symptom was defined as 0 for urgency and incontinence and as ≤7 for micturition frequency. For nocturia it was defined as 0 or as ≤1.

	Control	Diabetes	Hypertension	Combination
Urgency	30.8	18.5	26.5	18.0
Incontinence	52.5	42.3	59.9	34.7
Frequency	47.2	47.8	41.5	40.3
Nocturia (0)	18.8	9.5	10.9	8.2
Nocturia (≤1)	63.4	48.5	55.4	48.5

After 12 weeks of treatment, the median PPBC score had declined from 5 to 2 [2; 3] in control, to 3 [2; 3] in diabetic, to 2 [2; 3] in hypertensive, and to 3 [2; 4] in those with both comorbidities. Its distribution was similar in all groups (Figure 1). Moreover, shifts from baseline to 12 weeks were similar in all groups (Figure 1).

#### 3.1.4 Study II

**FIGURE 4:**
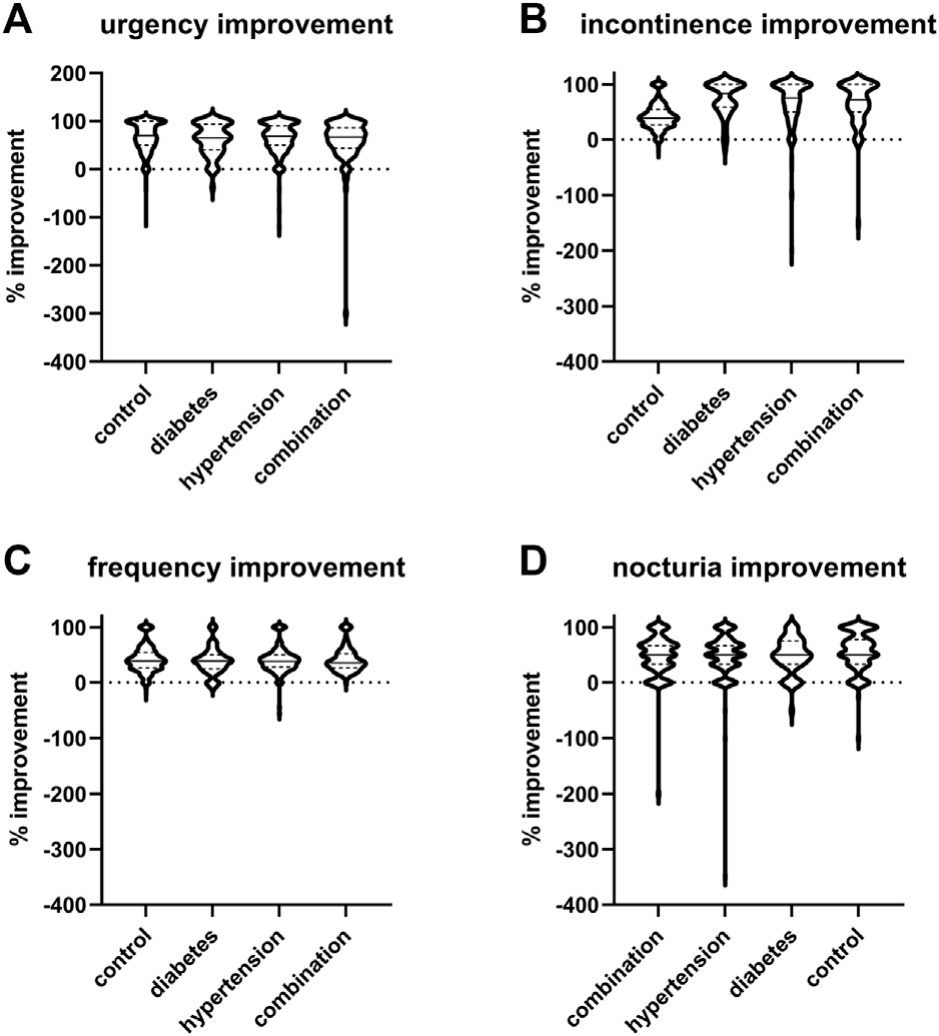
Violin plots of % changes in study II in number of **(A)** urgency, **(B)** incontinence, **(C)** frequency, and **(D)** nocturia episodes per 24 h. Median and interquartile ranges are indicated by the horizontal solid and dashed lines, respectively, within each plot. Note that only subject with a pathological value at baseline were included a very small number of subjects exhibited a worsening, indicated here as negative value.

The median and mean reductions in urgency, incontinence, and urgency episodes and in daily micturitions in study II (Figure 4) was comparable with that observed in study I (Figure 3). Thus, the mean reduction in urgency episodes was 66.5% ± 31.9% in control patients, 61.4% ± 33.2% in diabetic patients, 63.7% ± 33.3% in hypertensive patients, and 58.8% ± 47.0% in those with both comorbidities (median values with interquartile ranges shown in Figure 4). Similarly, the mean reduction in incontinence episodes was 69.6% ± 37.6% in control patients, 72.9% ± 31.1% in diabetic patients, 66.7% ± 43.6% in hypertensive patients, and 66.0% ± 39.2% in those with both comorbidities. The mean reduction in micturitions was 42.0% ± 23.6% in control patients, 38.3% ± 24.1% in diabetic patients, 40.4% ± 23.1% in hypertensive patients, and 41.7% ± 22.3% in those with both comorbidities (Figure 4). Finally, the mean reduction in nocturia episodes was 55.9% ± 34.1% in control patients, 48.8% ± 32.0% in diabetic patients, 49.2% ± 41.5% in hypertensive patients, and 48.1% ± 38.1% in those with both comorbidities. Becoming free of urgency was reported most often in control (27.0%), less often in hypertension and least in diabetes or combination (Table 4). Becoming free of incontinence and nocturia (control: 45.8% and 12.0%, respectively) exhibited a similar picture. Becoming free of frequency was similarly often in control (43.8%) and in patients with hypertension, but lower in those with diabetes or both comorbidities.

**TABLE 4:** Patients becoming free of a given symptom upon treatment in study II Data are shown as % of patients who had exhibited a symptom at baseline but did not at end of study. Not exhibiting a symptom was defined as 0 for urgency and incontinence and as ≤7 for micturition frequency. For nocturia it was defined as 0 or as ≤1.

	Control	Diabetes	Hypertension	Combination
Urgency	27.0	21.0	20.8	17.1
Incontinence	45.8	42.9	38.4	36.7
Frequency	43.8	33.3	41.5	34.0
Nocturia (0)	12.0	3.6	4.5	4.0
Nocturia (≤1)	57.3	34.0	49.2	37.9

After 12 weeks of treatment, the median PPBC score had declined from 5 to 2 [2; 3] in control, to 3 [3; 4] in diabetic, to 3 [2; 4] in hypertensive, and to 3 [2; 4] in those with both comorbidities. Its distribution was similar in all groups (Figure 2). Moreover, shifts from baseline to 12 weeks were similar in all groups (Figure 2).

## 4 Discussion

To the best of our knowledge, this is the first study exploring how the concomitant presence of diabetes and hypertension affects baseline symptom severity in OAB patients. Secondary research questions were how each condition alone affects baseline symptom severity and how each condition alone and their combination affect treatment outcomes.

### 4.1 Critique of methods

Our analyses are based on published data from two non-interventional studies (Amiri et al., 2021). The use of such observational data has intrinsic advantages and limitations. A potential disadvantage may be that the participating physicians were not provided with specific instructions how to diagnose the presence of concomitant diabetes and hypertension. Similarly, it has not been captured whether type 1 or 2 diabetes was present, but previous work indicates that this differentiation may, if anything, link to the prevalence of OAB whereas no difference in severity has been reported (Kempler et al., 2011). These limitations were required for the overall character of a non-interventional study but may have led to over- or underdiagnosis of comorbidities. Similarly, information on the duration of the comorbidities would have been of interest, but that had not been captured in the case record form to maintain the non-interventional character of the studies. The fact that number of reported comedications was considerably lower than what would be expected in a German population of this age (Selke Krulichová et al., 2021), indicates that comedication, and by inference also comorbidities may have been captured sub-optimally. The advantage is that use of data from non-interventional studies provides real-world evidence. This is important because for instance randomized controlled trials often have a long list of in- and exclusion criteria, which limit extrapolation to the general population, particularly regarding comorbidities. Of note, our study is based on patients seeking treatment for OAB symptoms, i.e., is not a population-based study. Therefore, it does not allow direct conclusions on the frequency of comorbidities. Accordingly, our analyses were limited to the question how the comorbidities of diabetes, hypertension and their combination are related to symptom intensity at the time of presentation.

Our data are based on a *post-hoc* analysis that had not been envisioned when the studies were planned and conducted. However, it is a strength of our data that they were based on a protocol that had been written, published, and approved by an additional ethical committee before data related to the present study questions had been observed. Pre-specification of analyses is a criterion to identify studies with a high probability to be reproducible (Vollert et al., 2020; Vollert et al., 2022).

Baseline and outcome data on OAB symptoms are often reported as means, which implicitly assumes that they exhibit a normal distribution. However, the assumption of a normal distribution appears untrue (Amiri et al., 2020). Therefore, our prespecified analysis protocol had specified to report data as medians with inter-quartile ranges (which does not assume a normal distribution); however, to enable comparability with previously published data, means ± SD are also reported.

It had also been specified in the published protocol for the present analyses that they were exploratory in nature. Therefore, in line with recent recommendations from leading statisticians (Amrhein et al., 2019; Michel et al., 2020b), no hypothesis-testing *p*-values were calculated. Moreover, in line with the non-interventional character of the underlying studies and the focus on real-world-evidence, we had not been interested to test whether certain associations occurred to a greater extent of any magnitude than expected based on chance alone, i.e., were statistically significant. As even small differences of limited medical relevance can become statistically significant in large datasets, we were rather interested in the question whether the comorbidities of diabetes, hypertension and their combination affected measured parameters to a clinically relevant extent. To compensate for the lack of hypothesis testing, we have concomitantly analyzed data from two studies of similar design, which increases the robustness of our findings. These strengths and limitations should be considered in the interpretation of our data.

### 4.2 Baseline data

The four groups differed in age in each of the two studies. While the diabetic groups were by average 6-7 years older than controls, hypertensives were another 1-2 years older, and the combination group was oldest. Previous non-interventional studies had also reported that OAB patients with concomitant diabetes (Schneider et al., 2013) or hypertension (Michel et al., 2022) were older than those without. The age differences associated with comorbidities reported here are quantitatively similar to those in the previous studies. There were also minor differences in gender distribution across the four groups of our studies. However, other non-interventional studies found that age and gender have only a small impact on baseline symptom severity in multivariate analyses (Michel et al., 2002; Schneider et al., 2010). The present groups with comorbidities also had a slightly greater BMI than the control groups, an expected feature particularly of T2DM populations. However, previous large studies had found that differences in BMI also do not affect baseline symptoms or treatment outcomes to a clinically relevant extent (Schneider et al., 2010; 2013). Therefore, no age-, gender- or BMI-adjusted analyses were deemed necessary and, for this reason, not implemented in our prespecified analysis protocol.

OAB patients with diabetes or hypertension reported more comedications than controls, and the combination group had more than either the diabetes or hypertension group. While this is highly plausible in principle, it is noteworthy that the median and mean number of 0 and <0.4 comedications, respectively, in the control group was surprisingly small for the age group under investigation. Recent analyses based on prescribing data in 6 million elderly individuals in Germany found that the average person aged ≥65 years used a median number of 7 medicines and only 6.5% did not receive any medication (Selke Krulichová et al., 2021). Thus, the overall number of concomitant medications apparently was under-reported, a consistent phenomenon of non-interventional studies including those with a large fraction with concomitant diabetes and/or hypertension (Michel et al., 1998; Michel et al., 2004a; Michel et al., 2008; Schneider et al., 2013). However, in line with all of the above reports, the present studies also found that number of comedications increased in the presence of comorbidities. Taken together and with additional consideration of the exploratory nature of our analyses, these findings justify that the analyses are based on univariate analysis.

Previous non-interventional studies had reported that OAB patients with concomitant diabetes (Schneider et al., 2013) or hypertension (Michel et al., 2022) had more severe symptoms than those without. This observation had more often been made in male populations with LUTS suggestive of BPH (Michel et al., 2000; Joseph et al., 2003; Michel et al., 2004b; Rohrmann et al., 2005; Gupta et al., 2006; Ponholzer et al., 2006; Martin et al., 2011; Michel et al., 2020c). It was confirmed in the present studies (secondary study aim). However, the effect sizes for concomitant diabetes and hypertension were small in the present and the previous studies, for instance ≤1.5 urgency, incontinence, frequency or nocturia episodes per 24 h in the present analyses. Thus, OAB patients with concomitant diabetes or hypertension seeking treatment present with a slightly greater symptom intensity than control patients. The fact that all parameters exhibited similar data in the present as in the previous studies for the isolated comorbidity of diabetes or hypertension, validates our overall findings.

The primary research question of our analyses had been whether the concomitant presence of both comorbidities, diabetes and hypertension, is associated with a greater symptom intensity than either comorbidity alone. Consistent across both studies, the dual comorbidity was associated with a greater median number of urgency episodes than either comorbidity alone, accounting for 1-2 additional episodes per 24 h. Similar findings were made for incontinence and frequency in study I, but this was not confirmed in study II where incontinence episodes were as frequent in patients with only diabetes and those with diabetes and hypertension as comorbidity. While our data point to some degree of comorbidity dose-response relationship, they also indicate that the associations are too weak to enable consistent detection, perhaps except for urgency. An apparent exception of this is PPBC, which was almost identical across the four groups. A previous non-interventional study had also reported that concomitant diabetes had only little impact on patient-reported outcome scores at baseline (Schneider et al., 2013).

### 4.3 Treatment responses

While the primary research question of the present analyses had a pathophysiological focus, two of our secondary questions related to treatment impact. Previous studies had reported that concomitant diabetes (Schneider et al., 2013) or hypertension (Michel et al., 2022) were associated with a reduced treatment response to two other muscarinic receptor antagonists, darifenacin and solifenacin, respectively. The two present studies with propiverine confirmed these observations. However, it is a consistent finding of all four studies that the attenuation of treatment responses by concomitant diabetes or hypertension was small relative to the overall improvement upon treatment. For instance, the median % reduction in number of urgency episodes was 72.8% in the control as compared to 66.0%, 69.5% and 64.0% in those with concomitant diabetes, hypertension and their combination, respectively, in study I. Thus, comorbidity did not attenuate treatment responses by >10%. Whether an individual patient can detect differences of this magnitude is unclear. The fraction of patients becoming free of a given symptom may better reflect individual patient experience (Müderrisoglu et al., 2022). These fractions were also reduced by presence of a single comorbidity and, in most cases, to a greater extent if both comorbidities were present. However, the chance to become free of a given symptom remained considerable, particularly for incontinence and frequency.

### 4.4 Conclusion

We conclude that the comorbidities of diabetes and hypertension are associated with a greater intensity of symptoms as compared to controls in OAB patients seeking treatment. The combined presence of both comorbidities is associated even greater baseline symptoms. While these associations are pathophysiologically interesting, their extent is unlikely to have major impact on individual patients. From a treatment perspective it possibly is more relevant that presence of one or two comorbidities were associated with attenuated treatment responses, but the degree of attenuation was small. Considering that there are no specific treatments for lower urinary tract dysfunction with comorbidities such as diabetes (Erdogan et al., 2022), existing OAB medications remain the best treatment option even if comorbidities are present. Therefore, we consider the present findings to be of pathophysiological and pharmacological interest but propose that they do not justify changing existing paradigms for the clinical management of OAB.

## Data Availability

The raw data supporting the conclusion of this article will be made available by the authors, without undue reservation.
